# Different linkages in the long and short regions of the genomes of duck enteritis virus Clone-03 and VAC Strains

**DOI:** 10.1186/1743-422X-8-200

**Published:** 2011-05-02

**Authors:** Xiaoli Liu, Zongxi Han, Yuhao Shao, Yang Li, Huixin Li, Xiangang Kong, Shengwang Liu

**Affiliations:** 1 Division of Avian Infectious Diseases, National Key Laboratory of Veterinary Biotechnology, Harbin Veterinary Research Institute, the Chinese Academy of Agricultural Sciences, Harbin 150001, the People's Republic of China

## Abstract

**Background:**

Duck enteritis virus (DEV) is an unassigned member in the family *Herpesviridae*. To demonstrate further the evolutionary position of DEV in the family *Herpesviridae*, we have described a 42,897-bp fragment. We demonstrated novel genomic organization at one end of the long (L) region and in the entire short (S) region in the Clone-03 strain of DEV.

**Results:**

A 42,897-bp fragment located downstream of the *LOFR11 *gene was amplified from the Clone-03 strain of DEV by using 'targeted gene walking PCR'. Twenty-two open reading frames (ORFs) were predicted and determined in the following order: 5'*-LORF11-RLORF1*-*ORF1*-*ICP4*-*S1-S2-US1-US10-SORF3-US2-MDV091.5-like-US3-US4-US5-US6-US7-US8-ORFx-US1-S2-S1-ICP4 *-3'. This was different from that of the published VAC strain, both in the linkage of the L region and S region, and in the length of the US10 and US7 proteins. The *MDV091.5-like *gene, *ORFx *gene, *S1 *gene and *S2 *gene were first observed in the DEV genome. The lengths of DEV US10 and US7 were determined to be 311 and 371 amino acids, respectively, in the Clone-03 strain of DEV, and these were different from those of other strains. The comparison of genomic organization in the fragment studied herein with those of other herpesviruses showed that DEV possesses some unique characteristics, such as the duplicated US1 at each end of the US region, and the US5, which showed no homology with those of other herpesviruses. In addition, the results of phylogenetic analysis of ORFs in the represented fragment indicated that DEV is closest to its counterparts VZV (*Varicellovirus*) and other avian herpesviruses.

**Conclusion:**

The molecular characteristics of the 42,897-bp fragment of Clone-03 have been found to be different from those of the VAC strain. The phylogenetic analysis of genes in this region showed that DEV should be a separate member of the subfamily *Alphaherpesvirinae*.

## Background

Herpesviruses are among the most persistent of all pathogens because they have coevolved with their hosts over a long period of time, and they are relatively harmless in immunocompetent hosts [1]. The family *Herpesviridae *comprises approximately 100 members; these viruses infect a range of host species from humans and other mammals to birds, amphibians, and reptiles [2]. On the basis of differences in cellular tropism, genome organization, and gene content, herpesviruses have been grouped into three subfamilies: *Alphaherpesvirinae *(α-), *Betaherpesvirinae *(β-), and *Gammaherpesvirinae *(γ-) [3,4]. Currently, duck enteritis virus (DEV), also known as duck plague virus (DPV) and duck herpesvirus-1 [4], is an unassigned member of the family *Herpesviridae *[5].

Herpesviruses are enveloped viruses with a virion size over 100 nm [1]. The genomes of these viruses are linear, double-stranded DNA, and they differ in size, sequence arrangements, and base composition [2]. They also vary significantly with respect to the presence and arrangement of inverted and directly repeated sequences [6]. Herpesvirus genomes differ in the arrangement of direct and inverted repeat regions with respect to unique regions. Six types of genome structures have been confirmed adequately in herpesviruses, which are designated by letters from A to F. The A type structure consists of a unique region flanked by a direct terminal repeat at the genome ends. Type B genomes contain variable numbers of a TR (terminal reiterations) at each end of the genome. In the C type genome, the number of direct terminal reiterations is small but sequences longer than 100 bp are directly repeated and subdivide the unique sequence of the genome into several well delineated stretches. The D type genome just has the repeated sequences at one terminus and in an inverted orientation internally. In the E group, the genome is divided into unique long (UL) and unique short (US) regions; each unique region is flanked by the inverted repeats. The sequences at the two termini of the F group are not identical and are not repeated directly or in an inverted orientation. It has been reported that DEV also contains linear, double-stranded DNA, and its genome was shown to be approximately 180 kb in size, with a G plus C content of 64.3% [7]. Genomic sequences of DEV have been reported recently by several Chinese research groups; however, discrepancies were found among these reports [8-18]. Genes in the UL region of DEV and their arrangement have been reported by our laboratory, and the results generally showed more similarity with *Mardiviruses *[8-13]. Another report showed that the *LORF11 *gene of the VAC strain is located at the leftmost end of the DEV genome, and that the *LORF11 *gene encoded a putative protein of 275 amino acids in the VAC strain [14]; both of these results differ from our previous results [12]. Meanwhile, several genes in the US region have also been reported [15-18]; however, the length of the putative proteins encoded by the *US10 *gene and *US7 *gene has been debated. In this study, we present a fragment of 42,897 bp, which contains one end of the L region that includes part of the *LORF11 *gene, which was absent from the published VAC strain, and the whole of the DEV S region. In addition, we demonstrated a different genomic organization of the junction of the L region and the S region in this study. These results will provide a useful comparative dataset for the study of related genes in DEV and other herpesviruses.

## Results

### The features of the overall sequences and determination of ORFs

**Figure 1:**
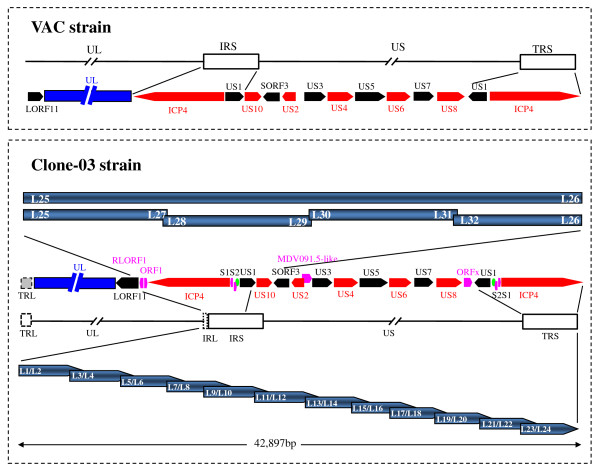
**The comparison of genomic organization between the DEV Clone-03 strain and the published VAC strain, together with the PCR strategy**. The upper part shows the genomic organization of sequences corresponding to those in the present study in the published sequences of the genome of the DEV VAC strain. The genomic organization of the presented fragment in the DEV Clone-03 is listed below. The red and blank arrows indicate the ORFs, with different colours to make two adjacent ORFs evident. The pink arrows indicate the ORFs that were not detected in other heprsviruses but were detected in DEV. The dark blue indicates the UL region; the dashed gray boxes in the genome of DEV Clone-03 indicate uncertain regions. The PCR strategy used to obtain the fragment is depicted at the bottom. The blue arrows indicate the twelve overlapping fragments that were obtained to form a continuous DNA fragment; the primer positions and amplification directions are also shown. The arrows indicate the genes and the interval shows the relative position of the two adjacent ORFs. The confirmation PCR strategy is depicted in the centre. The bars indicate the PCR product with the primers embedded. The green ellipse indicates the predicted origins of replication (oriS).

A fragment of 42,897 bp downstream of the *LORF11 *gene was amplified from the genome of the Clone-03 strain of DEV in this study. The genome structure and the gene layout of this fragment are depicted in Figure 1. The fragment contained part of the sequence of the *LORF11 *gene [12], the rightmost part of the L region, the US region and its flanking sequences, and inverted repeats of the short region (IRS and TRS). The L region and IRS were interrupted by a set of tandem repeat sequences designated as α-type-like sequences [13], as in the case of the two regions in herpes simplex virus (HSV) [19]. Another α-type-like sequence was also found at the end of the TRS in the DEV genome. The overall G plus C ratio of the region sequenced was 46.09%.

Twenty-two ORFs that contained more than 75 amino acids were found in the present study, which were in the order: 5'*-LORF11-RLORF1*-*ORF1*-*ICP4*-*S1-S2-US1-US10-SORF3-US2-MDV091.5-like-US3-US4-US5-US6-US7-US8-ORFx-US1-S2-S1-ICP4*-3'. These ORFs were predicted to encode 17 putative proteins, with the exception of *LORF11*, because genes in the IRS and TRS were inverted and encoded the same proteins. The start locations of all ORFs were assumed to be the first possible ATG. The motifs of each ORF are listed in Table 1.

**Table 1:** Core promoters searched in the neural network and polyadenylation signals predicted by POLYADQ

Gene	Promoter location^a^	Promoter score	TATA sequence	TATA location	Poly(A) sequence	Poly(A) location	Poly(A) score
*RLORF1*	NP^b^	NP	NP	NP	NP	NP	NP
*ORF1*	5,564-5,613	0.93	ATATAAAGCGGTAGT	5,575-5,589	NP	NP	NP
*ICP4*	10,928-10,977r^c^	0.88	TTTGTAAAAT	10,960-10,969r	AATAAA	5,867-5,872r	0.317475
*S1*	13,570-13,619r	0.85	CTATCTAAGGCGACC	13,602-13,611r	NP	NP	NP
*S2*	14,725-14,774	1.00	NP	NP	NP	NP	NP
*US1*	15,606-15,655	0.99	GCCTAAAAAGCACCG	15,613-15,628	AATAAA	17,015-17,020	0.644949
*US10*	17,003-17,052r	0.94	CAATAAACACCGCTT	17,014-17,028	NP	NP	NP
*SORF3*	19,280-19,329r	0.99	GCTTTAAAAG	19,313-19,322r	AATAAAr	18,248-18,253r	0.138679
*US2*	20,407-20,456r	0.85	GTCTAAAAGGCAGAG	20,434-20,448r	NP	NP	NP
*MDV091.5-like*	NP	NP	NP	NP	NP	NP	NP
*US3*	20,299-20,348	0.85	CCCATAAATG	20,305-20,314	NP	NP	NP
*US4*	21,799-21,848	0.96	GTATAAATTAGACAA	21,807-21,821	AATAAA	23,310-23,315	0.385382
*US5*	23,335-23,384	0.88	GTCTTGTGTTTATAT	23,335-23,349	AATAAA	25,170-25,175	0.266987
*US6*	25,058-25,107	0.94	CGGCAATATGTATAT	25,062-25,076	NP	NP	NP
*US7*	26,158-26,207	0.92	ATATAATTACTACGC	26,167-26,181	NP	NP	NP
*US8*	NP	NP	NP	NP	NP	NP	NP
*ORFx*	29,414-29,463	0.93	GTATATTAGGCCGAC	29,421-29,435	NP	NP	NP
*US1*	31,386-31,435r	0.99	GCCTAAAAAGCACCG	31,413-31,427r	AATAAA	30,021-30,026	0.644949
*S2*	32,267-32,316r	1.00	NP	NP	NP	NP	NP
*S1*	33,422-33,471	0.85	CTATCTAAGGCGACC	33,431-33,445	NP	NP	NP
*ICP4*	36,064-36,113	0.88	TTTGTAAAAT	36,072-36,081	AATAAA	41,170-41,175	0.317475

^a ^the position is according to the sequence of the whole fragment of 42,897 bp.

^b ^NP indicates no prediction.

^c ^r indicates reverse direction.

### The confirmation of the junction between the L region and the S region

Owing to the different linkages of the L region and S region found in the genome sequences of the published DEV VAC strain [14] and our above-described sequence in the Clone-03 strain of DEV, a pair of specific primers was designed to confirm the junction of the L region and S region in the DEV genome. The forward primer, L25, was located in the *LORF11 *gene (GenBank no. EU294364), which is a gene in the DEV UL region that had only one copy in the genome compared with the genomes of other alphaherpesviruses. The reverse primer, L26, was located in the *SORF3 *gene, which is a gene in the US region of the DEV genome that also has a single copy in the DEV genome. The PCR product was used as the model for the second nested PCR after dilution to 1 in 1,000. We obtained four different fragments (Figure 1), and they were 4,553 bp, 4,689 bp, 4,743 bp, and 5,547 bp in length, respectively. The results of sequencing of the four fragments showed that they were parallel with the sequences obtained using 'targeted gene walking PCR'. Consequently, we determined that the linkage between the L region and the S region should be in the following order: 5'*-LORF11-RLORF1*-*ORF1*-*ICP4 *-*S1-S2-US1-US10-SORF3*-3'.

### A 207-bp insertion in both the IRS and the TRS regions was not found in their counterparts in the DEV VAC strain

**Figure 2:**
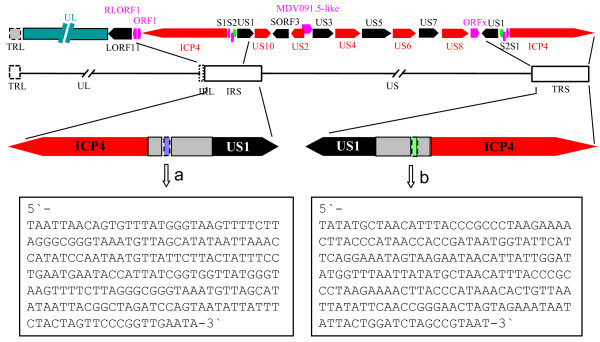
**The position of the two 207-bp insertions in the IRS and TRS regions in DEV Clone-03 strain comparing to the VAC strain**. The red and blank arrows indicate the main genes in the IRS and TRS regions. The gray boxes indicate the interval between the ICP4 and US1 genes. The blue and green boxes with dashed lines indicate the 207-bp fragment that was deleted from the IRS and TRS regions in the published VAC strain, respectively. Letters a and b indicate the fragment in the IRS and TRS regions, respectively. The sequences of the two fragments are listed in the boxes at the bottom.

In addition to the linkage of the L region and the S region, two insertions of 207 bp were found in the presented fragment in both the IRS region and the TRS region (Figure 2), when compared with the published VAC genome. The 158-bp sequence at the 3' end of the 207-bp sequence of the IRS region was complemented with a fragment of the same length at the 5' end of the 207-bp sequence of the TRS region. The remaining 49-bp fragment in each of the insertions was dissociated and not complemented. Both of the fragments were rich in A plus T, with a content of 67.15%.

### The characteristics of new ORFs detected in the fragment

Two ORFs, designated *RLORF1 *and *ORF1*, were detected in the region upstream of the S region. Another copy of *ORF1 *was found to the left of the DEV L region of the genome [13]. The *RLORF1 *and *ORF1 *encoded two putative proteins of 109 and 81 amino acids, respectively. Four phosphorylation sites were predicted in the sequence of RLORF1.

In addition, eight ORFs encoding four different putative proteins (S1, S2, ICP4, and US1) in the RS region were detected. Of these proteins, S1 and S2 were identified for the first time in the present study. The *S1 *gene encoded a putative protein of 92 amino acids, and four phosphorylation sites were predicted. No homologue of S1 was found in the proteins encoded by other herpesviruses. Another unique gene in the RS region was *S2*, which encoded a putative protein of 96 amino acids that contained just six phosphorylation sites. ICP4 and US1 were the same as previously described [13,14].

The DEV US region contained 11 ORFs that were likely to code for 11 proteins (Figure 1), which included homologues of the HSV-1 genes *US10*, *US2*, *US3*, *US4*, *US6*, *US7 *and *US8 *[20]. Interestingly, a unique ORF in the DEV US region, located downstream of *US8*, was predicted in the present study and named *ORFx*. The *ORFx *encoded a putative peptide of 118 amino acids. One transmembrane domain was detected in the ORFx between residue positions 95 and 115 at the N-terminus. Remarkably, the length of our DEV US10 was 311 amino acids, which was different from published results of 168, 169 and 298 amino acids [14,15,17]. We also found a sequence of 13 amino acids, CSFWCCLGHAATC (Additional file 1, Figure S1), which mapped to amino acids 236-248 and conformed to the C-C-H-C zinc finger motif as described in equine herpesvirus-1 (EHV-1) [21,22]

A new gene was predicted in this study, which was 327 bp in length and overlapped 197 bp at the 3'-terminus of the *US2 *gene. It was homologous to the proteins encoded by Marek's disease virus-1 (MDV-1), MDV-2 and HVT and was designated *MDV091.5-like *gene. BLAST searches using the amino acid sequence showed that this protein had some amino acid similarity with putative nucleotide-binding oligomerization domain-containing protein 2 of *Gasterostrus aculeatus*, the putative lyase of *Rhodococcus erythropolis*, and bacterial valyl-tRNA synthetase.

**Figure 3:**
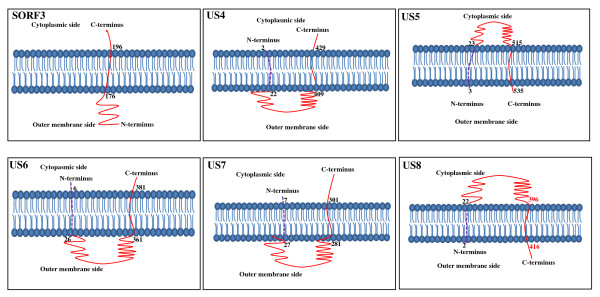
**Transmembrane regions in the proteins encoded by DEV genes in the present fragment**. The TMpred server was used to predict transmembrane regions. The dashed purple lines indicate the amino acid sequence of a potential signal peptide that may be cleaved during the maturation of proteins.

The transmembrane regions of the proteins encoded by the genes in the presented fragment are depicted in Figure 3. The conserved domains of US1, SORF3, US2, US3, US4, US6, US7, US8 proteins are shown in Additional file 2, Figure S2, Additional file 3, Figure S3, Additional file 4, Figure S4, Additional file 5, Figure S5, Additional file 6, Figure S6, Additional file 7, Figure S7, Additional file 8, Figure S8, Additional file 9, Figure S9, Additional file 10, Figure S10, respectively.

### Phylogenetic analysis

**Figure 4:**
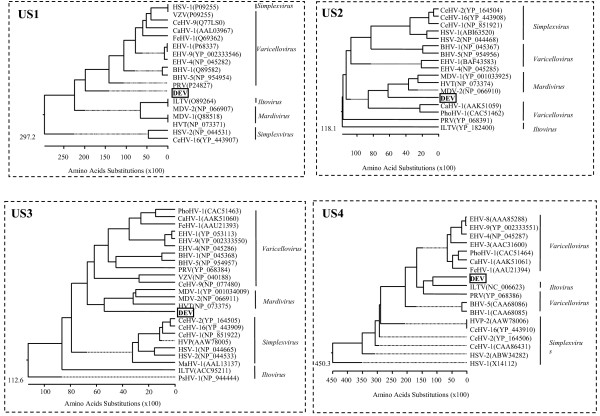
**Evolutionary relationship of US1, US2, US3 and US4 proteins in DEV S regions within the subfamily Alphaherpesvirinae**. The phylogenetic tree was generated using the MEGALIGN (DNAStar) program. Sequence distances indicated by the scale were calculated using the Gonnet 250 matrix in LASERGENE.

**Figure 5:**
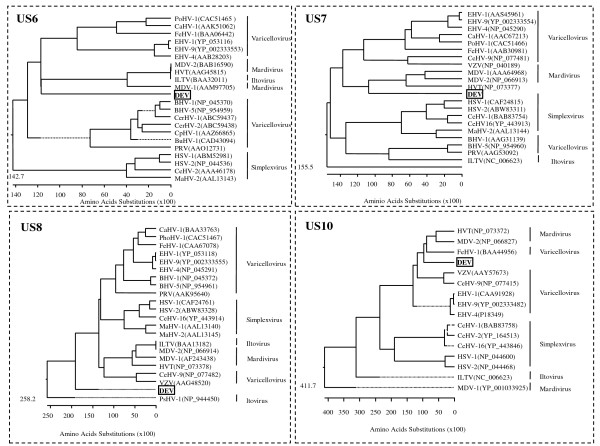
**Evolutionary relationship of US6, US7, US8 and US10 proteins in DEV S regions within the subfamily Alphaherpesvirinae**. The phylogenetic tree was generated using the MEGALIGN (DNAStar) program. Sequence distances indicated by the scale were calculated using the Gonnet 250 matrix in LASERGENE.

Phylogenetic rooted trees were constructed from alignments of the putative proteins with their homologues in other alphaherpesviruses and are shown in Figure 4 and 5. The DEV *US2 *gene, *US3 *gene, *US6 *gene, *US7 *gene and *US10 *gene showed closer relationships with members of *Mardivirus*. However, *US1 *showed a closer relationship between DEV and members of *Simplexvirus *and *Varicellovirus*. The DEV *US4 *gene showed more similarity with infectious laryngotracheitis virus (ILTV), and both clustered into the subfamily *Varicellovirus *(Figure 4). The DEV *US8 *gene fell into an outgroup position with respect to members of subfamily *Alphaherpesvirinae *(Figure 5), which implies that a recombination event may have occurred during the origin and evolution of the virus.

### The comparison of gene layouts in the US region of DEV with those in other alphaherpesviruses

**Figure 6:**
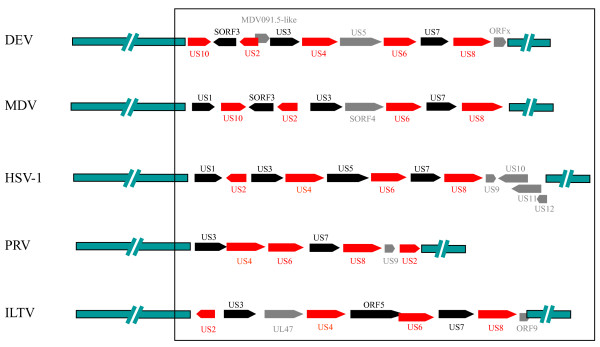
**Comparison of genes in the DEV and alphaherpesvirus US regions**. The comparison was based on the published sequences (37, 21, 20, 49). The red and blank arrows indicate the ORFs that were homologous in different alphaherpesviruses. The gray arrows indicate the ORFs that not exist in all the selected viruses. The green bars in the leftmost and rightmost parts indicate the sequences of the genomes, except for the US region. The targeted regions compared in this study are boxed.

A comparison of the genetic organization of selected alphaherpesvirus US segment genes is presented in Figure 6. Despite obvious similarities, there were marked differences in gene content, organization and localization between DEV and other alphaherpesviruses. Nevertheless, these overall gene layouts are consistent with a model that accounts for the divergence of alphaherpesvirus from a common ancestor by a number of homologous and semihomologous recombination events, which resulted in concomitant loss or gain of US genes [23].

### Origins of replication in the S region

**Figure 7:**
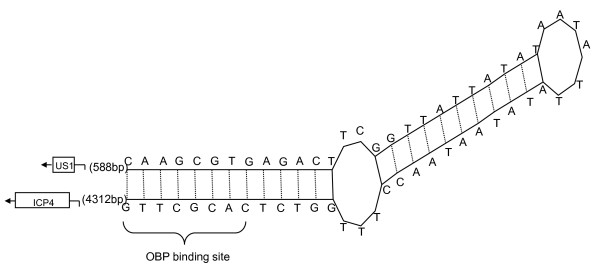
**Hairpin-like secondary structure reflecting palindromic nature of sequences of oriS**. The arrows and boxes at flanking the palindromes represented the two genes that flanked the oriS. The distances in nucleotides between the oriS sequence and the two genes are shown in brackets.

Two well-defined origins of replication were found in the IRS and TRS of the DEV genome, designated *oriS*. The two *oriS *were palindromic structures and contained the same sequence features: two inverted 9-bp sequences, which were identical to that recognized by the origin-binding protein (OBP) encoded by the *UL9 *binding sequence (GTTCGCAC), separated by a 43-bp AT-rich spacer sequence (76.75% A+T) (Figure 7). The features were the same as described for PRV (Pseudorabies virus) [24] and equine herpesvirus-1 (EHV-1) [25].

## Discussion

Our laboratory has been engaged for many years in analyzing the genome sequences of DEV [8-13]. After we had completed the genome sequence of DEV Clone-03, a DEV VAC genome sequence was also published by other researchers [14]. However, some differences were detected by comparison of parts of our DEV Clone-03 strain with those of the DEV VAC strain. Herein, we presented the sequence of a 42,897-bp fragment anchored in the *LORF11 *gene of the DEV genome which was located at the rightward end of the UL region [12], by using the method of 'targeted gene walking PCR' (Figure 1). Comparison of the sequence of the fragment with that of the DEV VAC strain showed that our Clone-03 strain of DEV had a different gene order from that of the DEV VAC strain in this region. Consequently, we designed an additional four pairs of primers according to the new sequences and confirmed the result using nested PCR (Figure 1). The two methods obtained the same sequences, and it was demonstrated that the genes in this region should be in the following order: 5'*-LORF11-RLORF1*-*ORF1*-*ICP4*-*S1-S2-US1-US10-SORF3*-3', which is different from the DEV VAC strain, in which the gene order is 5'*-LORF11-UL-ICP4-US1-US10-SORF3*-3' [14]. The different linkage pattern between DEV Clone-03 and the VAC strain in the L region and S region is difficult to explain and requires further investigation, although a different linkage between the L and S regions of HSV was also observed between wild-type virus and cell-adapted virus [26,27].

Interestingly, we also found some novel characteristics of the sequences in the S region of the Clone-03 strain of DEV. Two insertions of 207 bp in the IRS and TRS regions were found in the DEV Clone-03 strain that were absent from the VAC strain. It has been reported that some fragments were lost during serial passage of MDV [28]. Hence, we speculated that the insertion of the two 207-bp fragments in the DEV Clone-03 strain and their absence from the VAC strain might be due to the different passage levels [28]. The *S1 *gene, *S2 *gene, *RLORF1 *gene, *ORF1 *gene and *ORFx *gene that were observed in the Clone-03 strain in this study also had similar sequences in the VAC genome; however, those genes showed no homologues in other alphaherpesviruses. Those genes may be potential markers to differentiate DEV from other alphaherpesviruses.

Davison and McGeoch concluded that differences in gene layout in the S component between HSV-1 and VZV have resulted from expansion and contraction of IRS/TRS during evolution [23]. This may also be the case for the DEV genome. Unlike those of MDV-1, MDV-2 and HVT, the DEV *US1 *gene was duplicated and also inverted to the other end of the US, as is that of PRV [24]. Similarly, the presence of two copies of the *US1 *gene in DEV does not imply that the virus expresses two forms of ICP22 [24]. Although the pattern of the two copies of the *US1 *gene in the DEV genome showed a similar gene layout to those of PRV, the existence of the *LORF11 *gene at the rightward end of the UL region indicated that the organization of the DEV genome may be similar to that of other avian herpesviruses. The presence of the *SORF3 *gene and the *MDV091.5-like *gene, and the translocation of the *US10 *gene in the DEV genome, further suggests a close relationship between DEV and other avian herpesviruses. In addition, the phylogenetic analysis of most genes in the presented fragment further indicated a close relationship between DEV and viruses in the subfamily *Mardivirus*. However, the US region of DEV contained some genes that were absent from the genomes of other avian herpesviruses, such as *US4 *and *ORFx*, which indicates that DEV may be a unique member of the subfamily *Alphaherpesvirinae*.

Replication of the viral genome is a central event in the life cycle of herpesviruses. The initiation of viral DNA synthesis marks the commitment of the infected cell to the production of new infectious virus and, in most instances, cell death. HSV-1 contains three origins of DNA replication of two types: one copy of *oriL *located at the centre of the UL region of the genome and two copies of *oriS *located in the repeat regions that flank the US region of the genome [29]. The reasons for the three potential origins of replication in the viral genome are not apparent in HSV. In this study, we predicted two copies of *oriS *in the RS region of DEV. It has been reported that the deletion of the *oriL *in HSV resulted in reduced replication in mouse tissues and reduced reactivation from latent infection. Thus, *oriL *may be required for DNA replication in certain tissues [29]. Although *oriL *was absent from the DEV genome, the core sequence of *oriS*, which typically contains an origin recognition element and a DNA-unwinding element, was unchanged [29]. This absence of *oriL *from DEV may be associated with the evolution of the viral genome, may lead to different characteristics of the replication of DEV from those of other herpesviruses, and may even result in functional deletions from the genome of DEV in comparison with other herpesviruses.

## Conclusion

In this study, we demonstrated a different organization of genes in the rightward part of the L region and the whole S region in the Clone-03 strain of DEV, when compared with the VAC strain. Several novel characteristics were also detected in this region that have not been reported in the VAC strain, including the presence of *S1*, *S2*, *ORFx *and *MDV091.5-like *genes and two insertions in the IRS and TRS regions. The genomic order and the characteristics of the genes in this region, together with phylogenetic analysis based on the putative proteins encoded by the genes investigated in the present study showed that DEV should be a unique member of the subfamily *Alphaherpesvirinae*.

## Methods

### Virus stock preparation

The Clone-03 strain of DEV was used in this study [8-13]. The virus stocks were produced by propagation in chicken embryo fibroblasts (CEF) in Dulbecco's minimum essential medium (DMEM) with 8% fetal bovine serum. The infected CEFs were harvested when the cytopathic effect (CPE) reached 80%. After three freeze-thaw cycles, the virus stocks were confirmed primarily by electron microscopy and polymerase chain reaction (PCR) as described previously [8-13].

### DNA extraction, polymerase chain reaction and sequencing

The viral DNA was extracted from the virus stocks as described previously [9]. The 'targeted gene walking PCR', as described previously [30,31], was used to amplify the targeted DEV genome fragment, as illustrated in Figure 1. Briefly, four nonspecific 'walking' primers, N1, N2, N3 and N4 [10] were used to walk the genome of DEV. A pair of specific primers, L1 and L2, was designed on the basis of the partial sequence of DEV *LORF11 *published in GenBank (GenBank no. EU294364) [12]. The PCR was carried out by using L1 and L2 as forward primers; the four nonspecific primers were used as reverse primers. Finally, a 2,998-bp fragment (F1) was amplified, anchored from the DEV *LORF11 *gene. Targeted primer L3 and internal primer L4 were designed on the basis of the newly generated fragment, and was used to amplify the neighbouring gene fragment with one of the four nonspecific primers. Similarly, primers L5-L6, L7-L8, L9-L10, L11-L12, L13-L14, L15-L16, L17-L18, L19-L20, L21-L22 and L23-L24 (Figure 1) were designed and used in the subsequent PCR amplifications. The primers used in PCR amplifications in this study are listed in Table 2.

**Table 2:** Sequences of oligonucleotides primers used for PCR amplification

Primer	Sequences (5'-3')	Position^a^
L1	AGTCCAGTCATCTCCATCCG	1,562-1,581
L2	ACGATTTGGCTGTGCTGTAG	1,730-1,749
L3	CTGTCTTAAGGTTAGGGCTGGC	4,532-4,553
L4	GTAGGAAATATTGAGCCGAG	4,826-4,845
L5	TTCTGATGTTTTGGCAAGCC	7,578-7,597
L6	CAGAATGGCGCTTTGTTTGG	7,669-7,688
L7	TTGAAGATAGGTTGCTCGTAG	11,272-11,292
L8	ATACAGGAAAATTAACGAT	11,367-11,385
L9	ATGTAGCAGTTTGTTCAAAC	14,193-14,212
L10	AAGATGAGTCAACACCGAAGG	14,445-14,465
L11	TATTCCATCCAGTTGCTCCC	17,732-17,751
L12	CTTGTAAAGCTGGCCGCTAC	17,978-17,997
L13	CGATCTGCTTTCGCTTTCCG	21,725-21,744
L14	TAGCTGGTATGGCAACAATG	21,895-21,914
L15	GAAGTTAACGGAGGAAGTATT	26,043-26,063
L16	GCATATAATTACTACGCAACC	26,165-26,185
L17	GTCATCCTTGTTATGTTGA	29,697-29,715
L18	CCTACTTGGTGGTCGGCCA	29,795-29,813
L19	CAGGATTTGATAACTAACC	34,266-34,284
L20	ATAAGCGCACTAGATGGCAG	34,473-34,492
L21	TGAACGGACCTTTGCTAATGAC	37,793-37,813
L22	GAGGGGTGGTACTGGTTCCG	38,120-38,139
L23	CCTACAATAACCTGGGAACT	40,770-40,789
L24	GATCTTGTCCGATGGGGATG	40,949-40,968
L25	ATGGGACAGTCCCTACCGTTGGCCTCGATTCAAAGCTTCTCAG	1-43
L26	GGACTGCAGGCCTTTCAACGGCCCCTGATATGGCTACTATGTC	18,716-18,758r^b^
L27	GCCAGCCCTAACCTTAAGACAG	4,532-4,553r
L28	CAACCCCGCCCACAATAAGC	4,043-4,062
L29	GCTTACATGCTTTTCCCCGC	8,712-8,731r
L30	CGAACCGTCACAGTCTGCAG	8,592-8,611
L31	GCCGCGCAGTAAGTACTCAG	13,315-13,334
L32	TGCCGATATCATTGGTTCAT	13,212-13,231
N1	TATAGGTTT(C/A)TGTT	NP^c^
N2	CTTTTGGAGCTG	NP
N3	GAATGTGA(A/G)AA	NP
N4	CATGTCTGCCGA	NP

^a ^the position is according to the sequence of the whole fragment of 42,897 bp.

^b ^r indicates the reverse direction.

^c ^NP indicates no prediction.

The PCR was carried out in a 25 μl reaction volume as described previously [9]. The reaction was performed at 95°C for 5 min, followed by 30 cycles of 94°C for 1 min, 50°C for 1 min and 72°C for 3 min; the reaction was ended by elongation at 72°C for 10 min. The PCR products were analyzed on a 0.8% agarose gel. The PCR products were sequenced directly or cloned into the pMD18-T vector (TaKaRa, Dalian, China) according to the manufacturer's instructions and used for sequencing. Each of the fragments was sequenced at least three times from different PCR products.

### The determination of open reading frames (ORFs) in the presented fragment and genomic organization in the junction between the L region and S region in the DEV genome

The sequences obtained were assembled using the Gene Runner (version 3.00, Hastings Software, Inc., Hudson, NY, USA). The ORFs and genomic organization in the junction of the L region and S region, and the layout of genes in the S region, were determined by comparison with the sequence counterparts of Marek's disease virus (MDV), HSV-1 and varicella-zoster virus (VZV). The same program was used to detect ORFs encoding proteins of greater than or equal to 75 amino acids with a methionine (M) start codon. The predicted ORFs and flanking sequences were evaluated for coding potential by detecting the promoter http://www.fruitfly.org/seq_tools/promoter.html[32], and the presence of TATA box http://motif.genome.jp/ and transcription terminal signals http://rulai.cshl.org/tools/polyadq/polyadq_form.html. Searches of the deduced proteins for signal peptides http://www.cbs.dtu.dk/services/SignalP/, transmembrane regions http://www.ch.embnet.org/software/TMPRED_form.html, N-linked glycosylation sites http://www.cbs.dtu.dk/services/NetNGlyc/ and serine, threonine and tyrosine phosphorylation sites http://www.cbs.dtu.dk/services/NetPhos/ were also performed online. The secondary structure of sequences in the *oriS *was constructed by using GeneQuest in DNAStar.

### Confirmation of the junction between the L region and the S region by specific PCR

Owing to the different order of genes in the junction of the L region and S region in the DEV Clone-03 in this study and the reported DEV VAC strain [14], one pair of specific primers, L25 and L26 (Table 2), was designed to confirm the result. Primer L25 was located within the *LORF11 *gene and L26 was located within the *SORF3 *gene. This pair of primers was used in the first nested PCR. Other primers, L27, L28-L29, L30-L31, and L32 (Table 2), were also used in the second nested PCR. The position of the primers and the strategy for confirmation of the sequence are shown in Figure 1.

The PCR was carried out in a 25 μl reaction volume. The first nest of the PCR reaction was performed at 95°C for 5 min, followed by 35 cycles of 94°C for 1 min, 50°C for 1 min and 72°C for 8 min; the reaction was ended by elongation at 72°C for 10 min. The PCR product was analyzed on a 0.8% agarose gel and was used as the template for the second nest. The second nested PCR was performed at 95°C for 5 min, followed by 30 cycles of 94°C for 1 min, 53°C for 1 min and 72°C for 3 min; the reaction was ended by elongation at 72°C for 10 min. The products of the second nested PCR were cloned and sequenced, respectively.

### Phylogenetic analysis

Homologue searches were conducted using BLAST searching [33] and phylogenetic analysis was performed using the MEGALIGN program in Lasergene (DNAStar) with CLUSTAL W multiple alignment and weight matrix Gonnet 250 [13]. The result was confirmed by use of the MAGE package (Version 4.0). The sequences of the herpesviruses that were used as reference strains for homology analysis were obtained from the GenBank database and the GenBank accession numbers are given in the phylogenetic trees.

### GenBank accession numbers

The DNA sequence of 42,897 bp from the DEV Clone-03 genome has been deposited in the GenBank database with the GenBank accession no. HQ009801.

## List of abbreviations used

DEV: duck enteritis virus; DVE: duck viral enteritis; DPV: duck plaque virus; ORF: open reading frame; L: long; S: short; UL: unique long; US: unique short; α: *Alphaherpesvirinae*; β: *Betaherpesvirinae*; γ: *Gammaherpesvirinae*; CEF: chicken embryo fibroblasts; DMEM: Dulbecco's minimum essential medium; CPE: cytopathic effect; PCR: polymerase chain reaction; MDV: marek's disease virus; HVT: turkey herpesvirus; VZV: varicella-zoster virus; PRV: pseudorabies virus; M: methionine; ILTV: infectious laryngotracheitis; EHV: equine herpesvirus; CeHV: cercopithecine herpesvirus; CaHV-1: canid herpesvirus-1.

## Competing interests

The authors declare that they have no competing interests.

## Authors' contributions

XL, SL and XK designed research; XL, ZH, YS and YL performed research; XL, SL, XK and HL analyzed data; and XL, SL and XK wrote the paper. All authors read and approved the final manuscript. 

## Supplementary Material

Additional file 1 **Figure S1: Multiple alignments of homologues based on US10 proteins of DEV Clone-03 and other typical strains of the subfamily *Alphaherpesvirinae***. The pink box indicate the probable C-C-H-C zinc finger motif in US10 proteins by comparison with their homologues in other herpesviruses. Click here for file

Additional file 2 **Figure S2: Multiple alignments of homologues based on US1 proteins of DEV Clone-03 and other typical strains of the subfamily *Alphaherpesvirinae***. Click here for file

Additional file 3 **Figure S3: Multiple alignments of homologues based on SORF3 proteins of DEV Clone-03 and other avian herpesviruses**. Click here for file

Additional file 4 **Figure S4: Multiple alignments of homologues based on US2 proteins of DEV Clone-03 and other typical strains of the subfamily *Alphaherpesvirinae***. The conserved domains were indicated by pink boxes. Click here for file

Additional file 5 **Figure S5: Multiple alignments of homologues based on the amino acid sequences in the N-terminus of US3 proteins of DEV Clone-03 and other typical strains of the subfamily *Alphaherpesvirinae***. The conserved domains (from I to VI) were indicated by pink boxes. Click here for file

Additional file 6 **Figure S6: Multiple alignments of homologues based on the amino acid sequences in the C-terminus of US3 proteins of DEV Clone-03 and other typical strains of the subfamily *Alphaherpesvirinae***. The conserved domains (from VII to XI) were indicated by pink boxes. Click here for file

Additional file 7 **Figure S7: Multiple alignments of homologues based on US4 proteins of DEV Clone-03 and other typical strains of the subfamily *Alphaherpesvirinae***. The conserved domains were indicated by pink boxes. Click here for file

Additional file 8 **Figure S8: Multiple alignments of homologues based on US6 proteins of DEV Clone-03 and other typical strains of the subfamily *Alphaherpesvirinae***. The conserved domains were indicated by pink boxes. Click here for file

Additional file 9 **Figure S9: Multiple alignments of homologues based on US7 proteins of DEV Clone-03 and other typical strains of the subfamily *Alphaherpesvirinae***. Click here for file

Additional file 10 **Figure S10: Multiple alignments of homologues based on US8 proteins of DEV Clone-03 and other typical strains of the subfamily *Alphaherpesvirinae***. The conserved domains were indicated by pink boxes. Click here for file
