# Supplementary figures and images for: Different linkages in the long and short regions of the genomes of duck enteritis virus Clone-03 and VAC Strains
Source: Virol J. 2011 May 2;8:200. doi: 10.1186/1743-422X-8-200 (PMC3113978; doi:10.1186/1743-422X-8-200)

## Slide 1
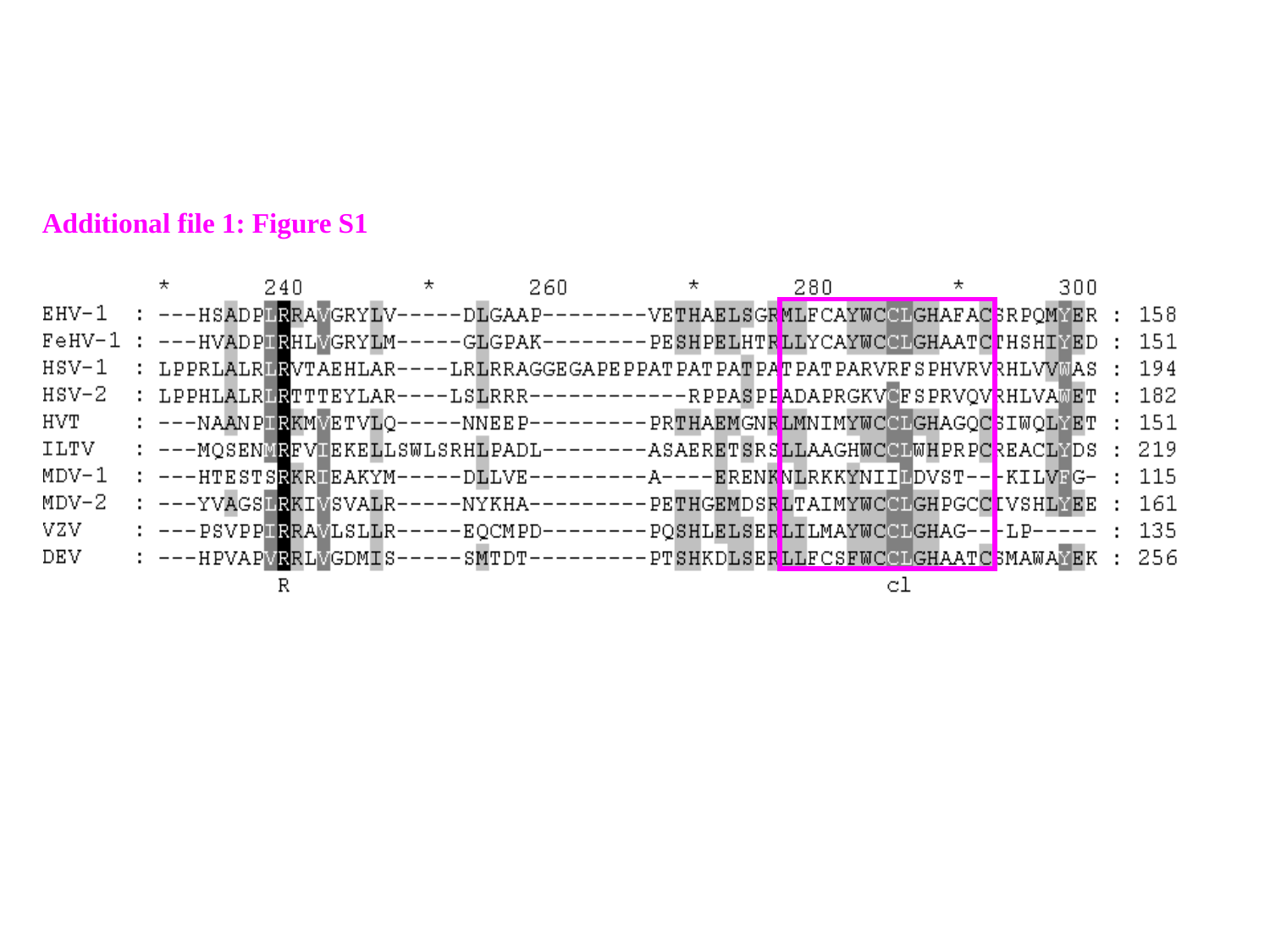

Additional file 1: Figure S1

Supplement: Additional file 1 — Figure S1: Multiple alignments of homologues based on US10 proteins of DEV Clone-03 and other typical strains of the subfamily Alphaherpesvirinae. The pink box indicate the probable C-C-H-C zinc finger motif in US10 proteins by comparison with their homologues in other herpesviruses. [file 1743-422X-8-200-S1.PPT]

## Slide 1
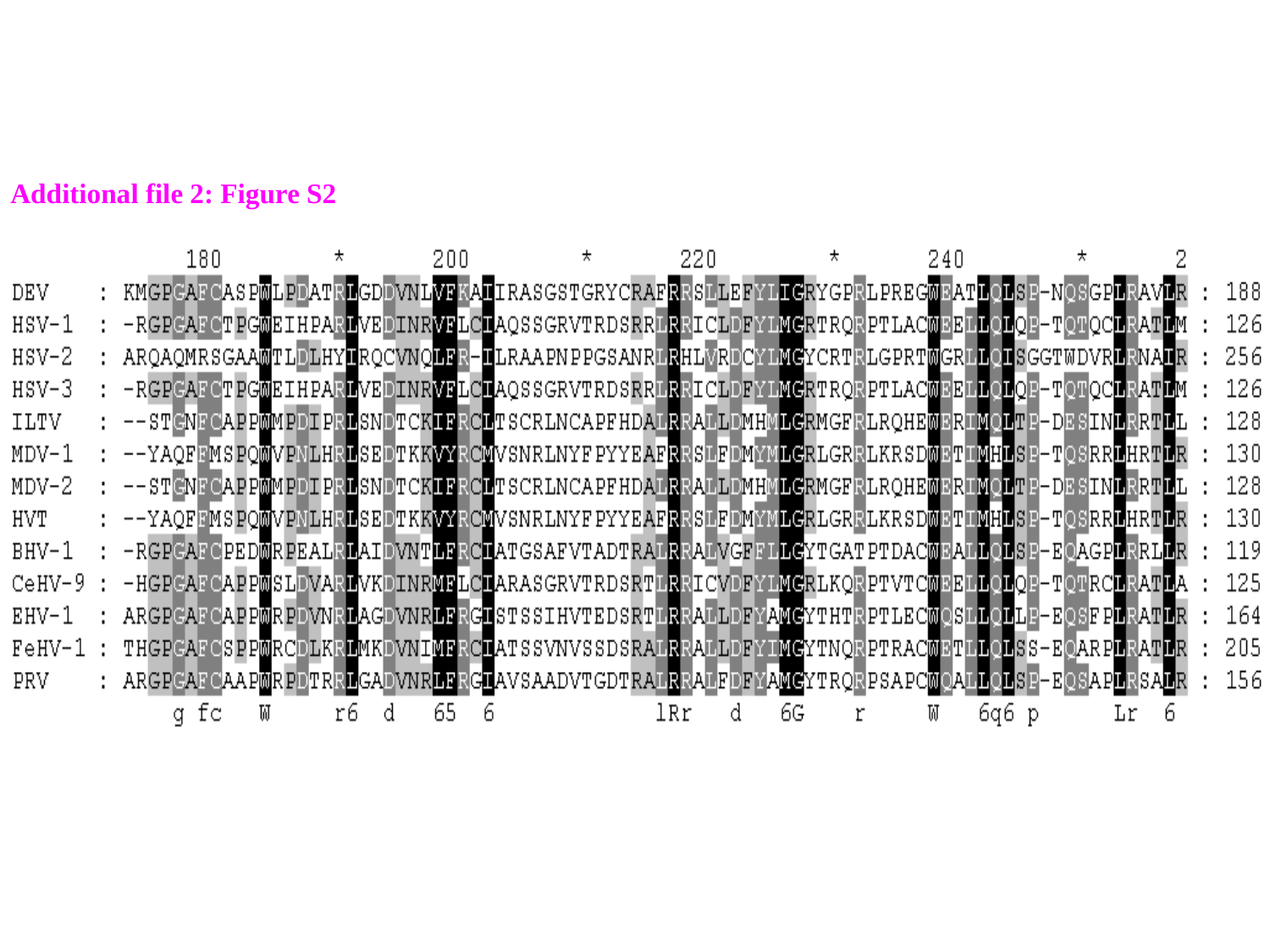

Additional file 2: Figure S2

Supplement: Additional file 2 — Figure S2: Multiple alignments of homologues based on US1 proteins of DEV Clone-03 and other typical strains of the subfamily Alphaherpesvirinae. [file 1743-422X-8-200-S2.PPT]

## Slide 1
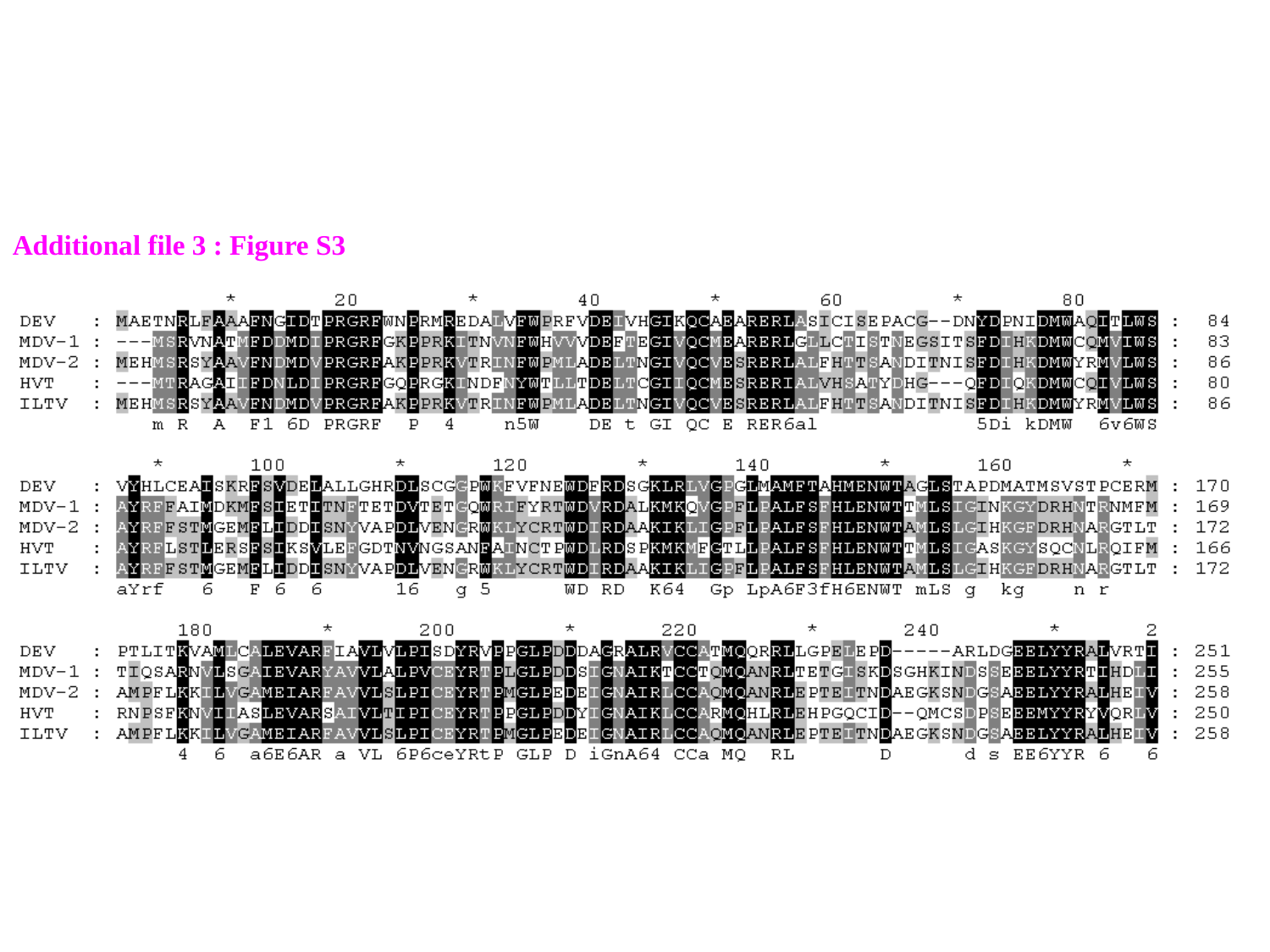

Additional file 3 : Figure S3

Supplement: Additional file 3 — Figure S3: Multiple alignments of homologues based on SORF3 proteins of DEV Clone-03 and other avian herpesviruses. [file 1743-422X-8-200-S3.PPT]

## Slide 1
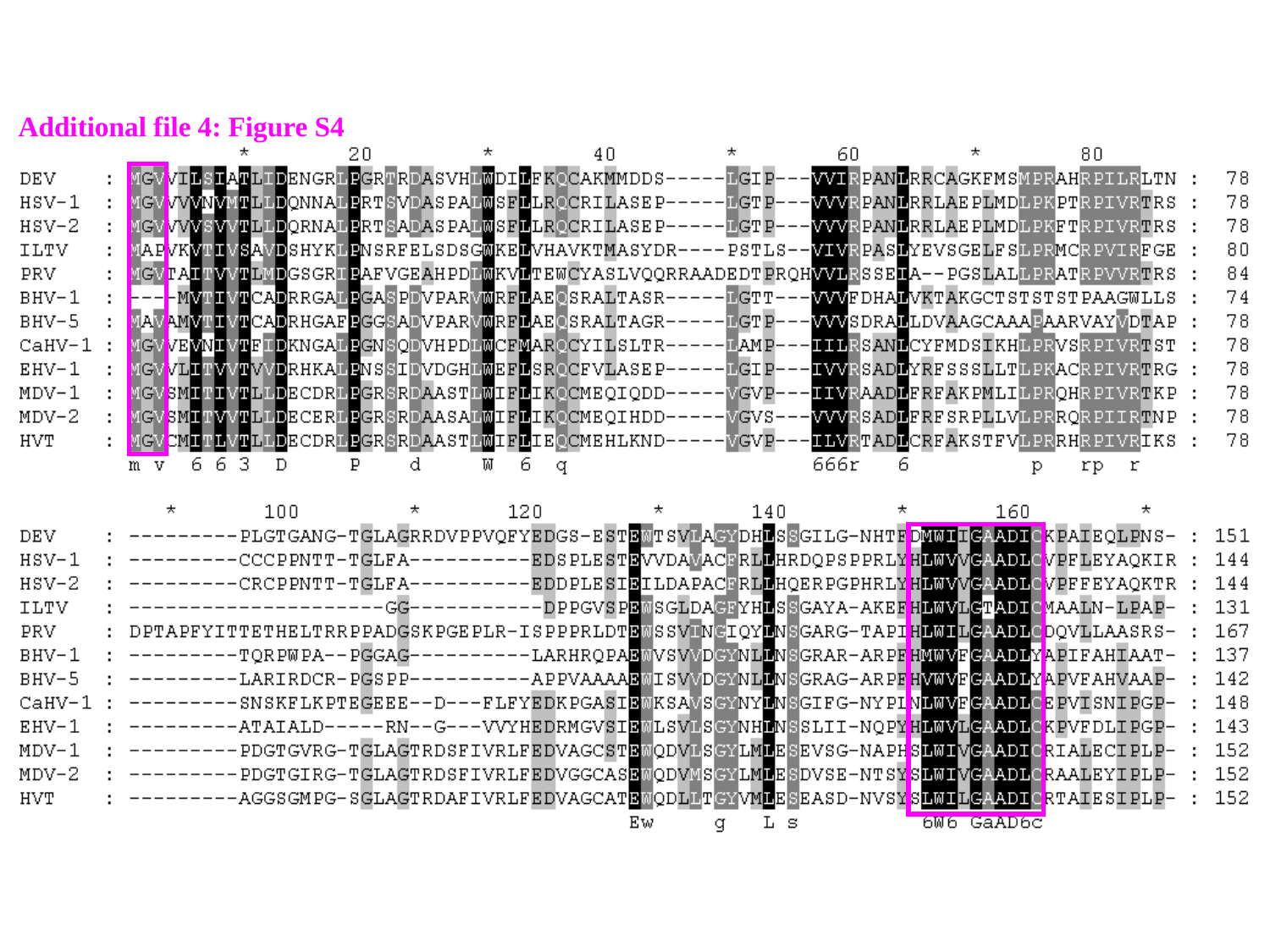

Additional file 4: Figure S4

Supplement: Additional file 4 — Figure S4: Multiple alignments of homologues based on US2 proteins of DEV Clone-03 and other typical strains of the subfamily Alphaherpesvirinae. The conserved domains were indicated by pink boxes. [file 1743-422X-8-200-S4.PPT]

## Slide 1
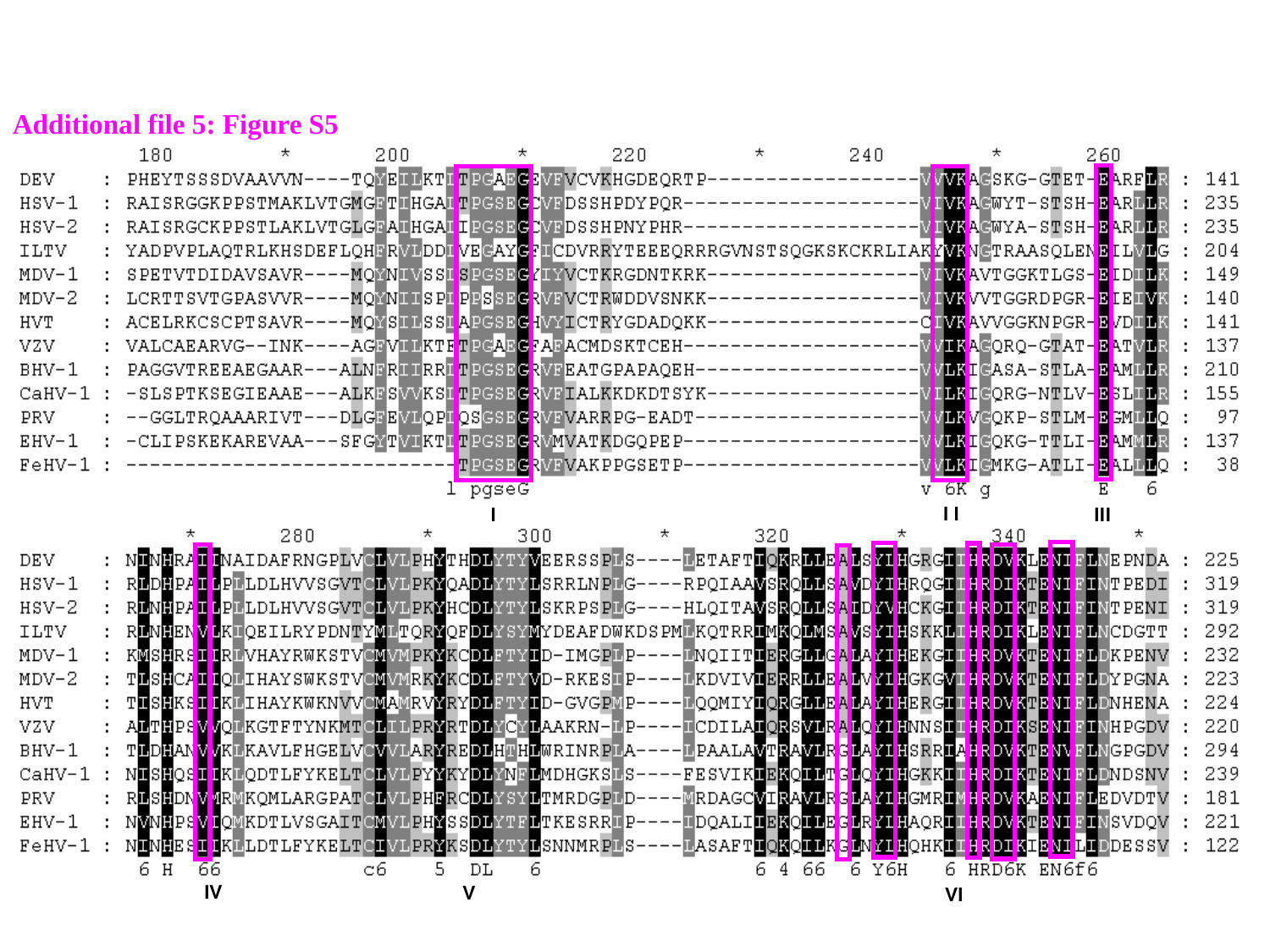

Additional file 5: Figure S5
I I
I
III
IV
V
VI

Supplement: Additional file 5 — Figure S5: Multiple alignments of homologues based on the amino acid sequences in the N-terminus of US3 proteins of DEV Clone-03 and other typical strains of the subfamily Alphaherpesvirinae. The conserved domains (from I to VI) were indicated by pink boxes. [file 1743-422X-8-200-S5.PPT]

## Slide 1
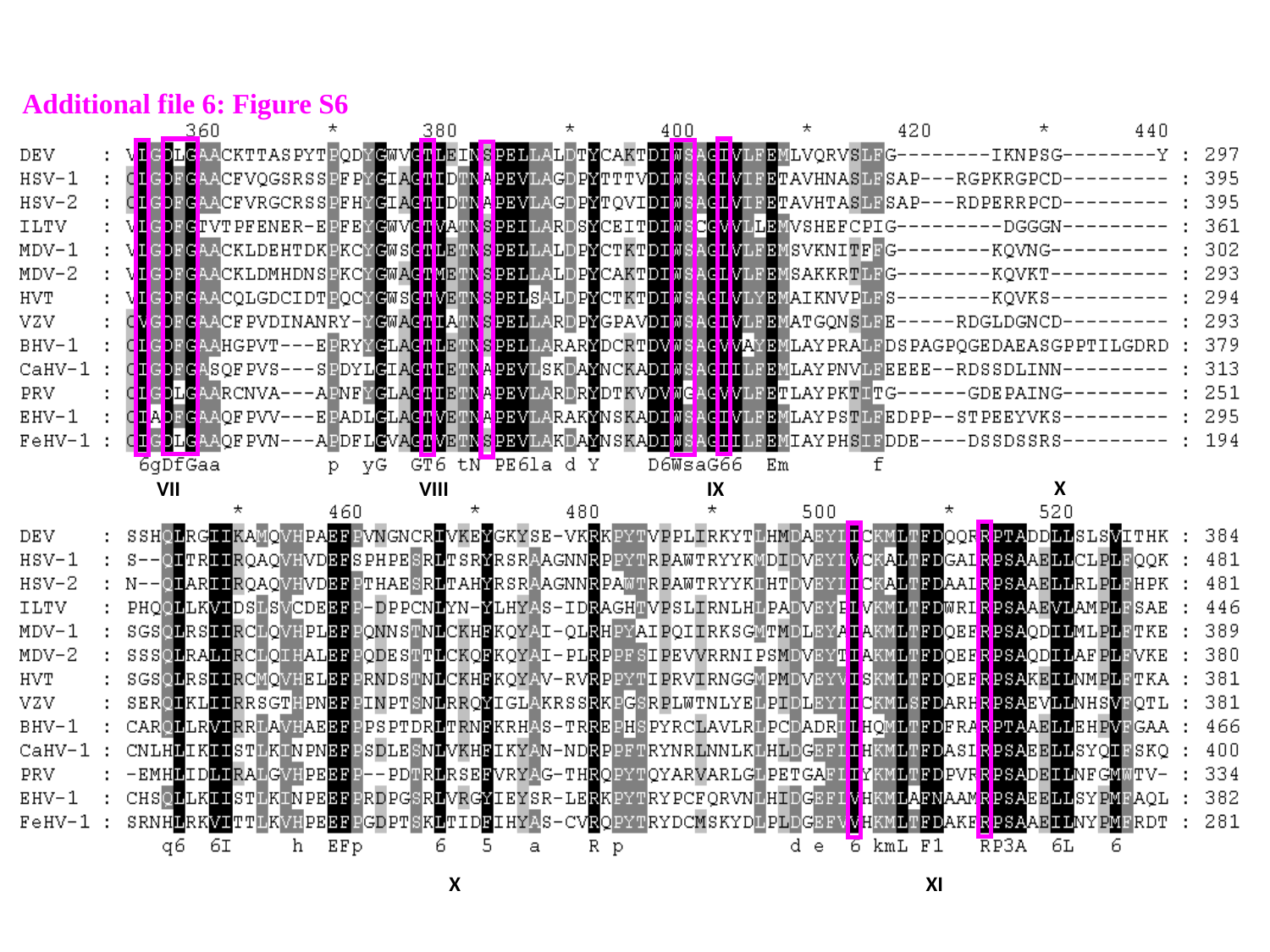

Additional file 6: Figure S6
X
VII
VIII
IX
X
XI

Supplement: Additional file 6 — Figure S6: Multiple alignments of homologues based on the amino acid sequences in the C-terminus of US3 proteins of DEV Clone-03 and other typical strains of the subfamily Alphaherpesvirinae. The conserved domains (from VII to XI) were indicated by pink boxes. [file 1743-422X-8-200-S6.PPT]

## Slide 1
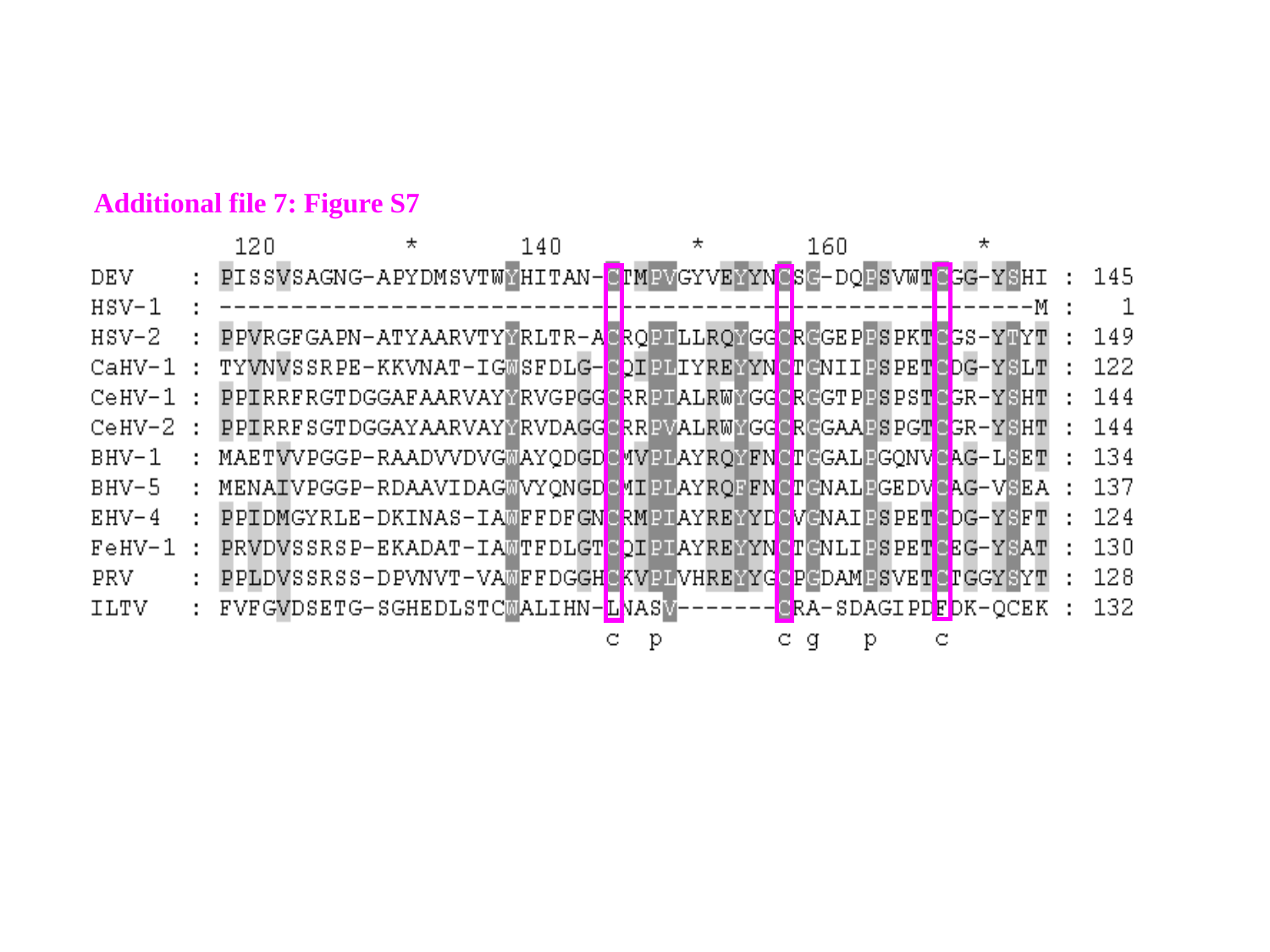

Additional file 7: Figure S7

Supplement: Additional file 7 — Figure S7: Multiple alignments of homologues based on US4 proteins of DEV Clone-03 and other typical strains of the subfamily Alphaherpesvirinae. The conserved domains were indicated by pink boxes. [file 1743-422X-8-200-S7.PPT]

## Slide 1
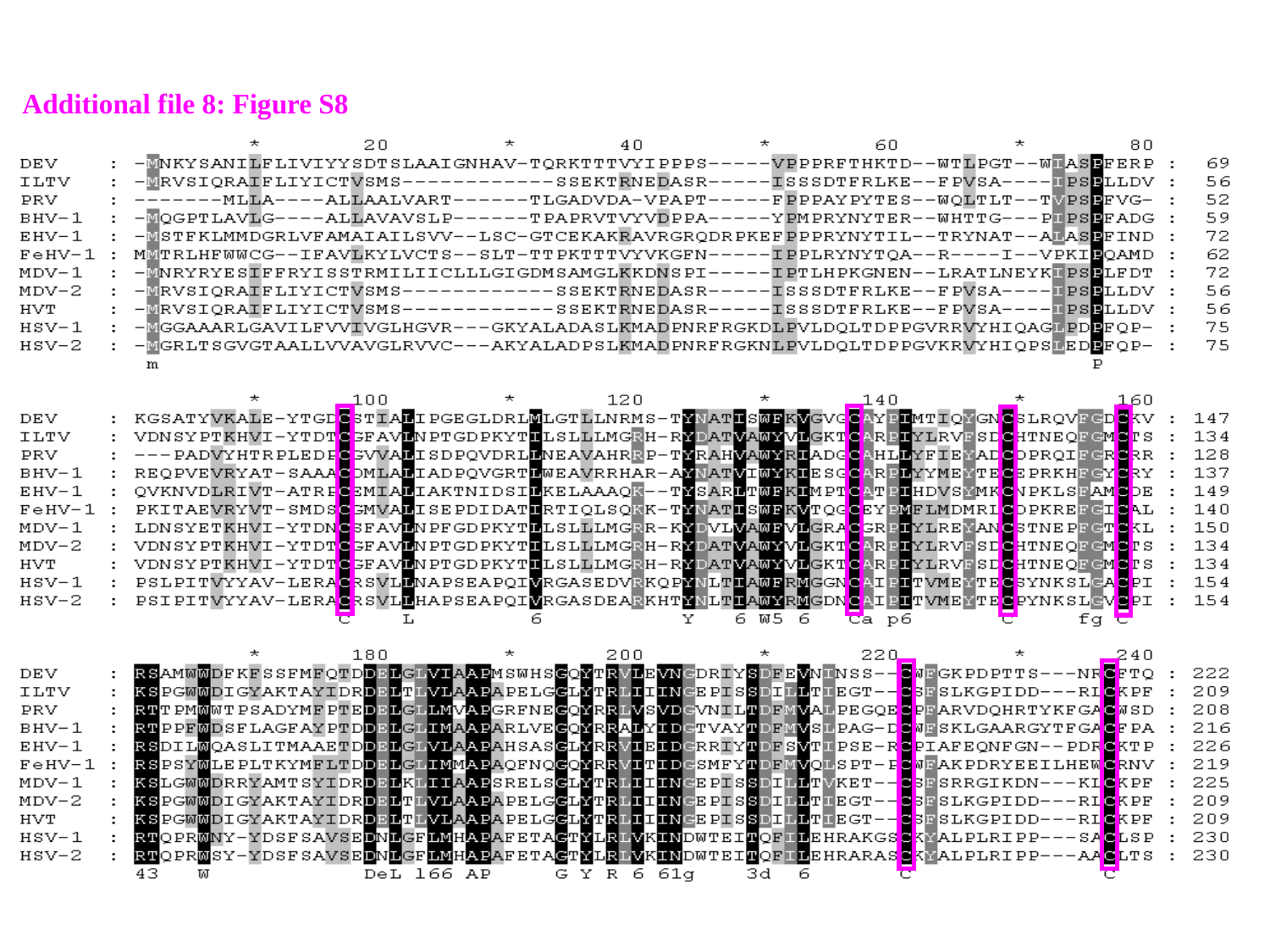

Additional file 8: Figure S8

Supplement: Additional file 8 — Figure S8: Multiple alignments of homologues based on US6 proteins of DEV Clone-03 and other typical strains of the subfamily Alphaherpesvirinae. The conserved domains were indicated by pink boxes. [file 1743-422X-8-200-S8.PPT]

## Slide 1
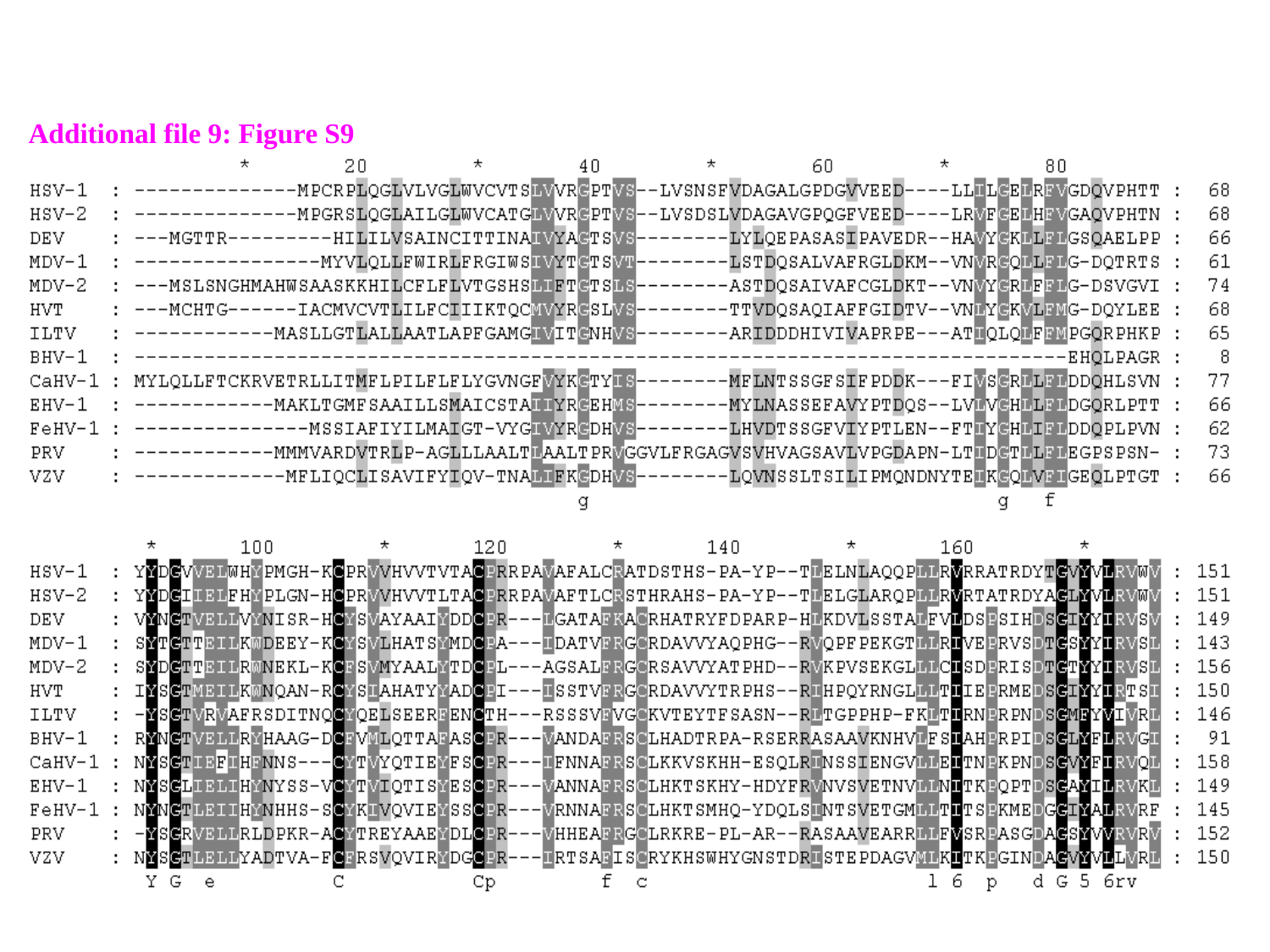

Additional file 9: Figure S9

Supplement: Additional file 9 — Figure S9: Multiple alignments of homologues based on US7 proteins of DEV Clone-03 and other typical strains of the subfamily Alphaherpesvirinae. [file 1743-422X-8-200-S9.PPT]

## Slide 1
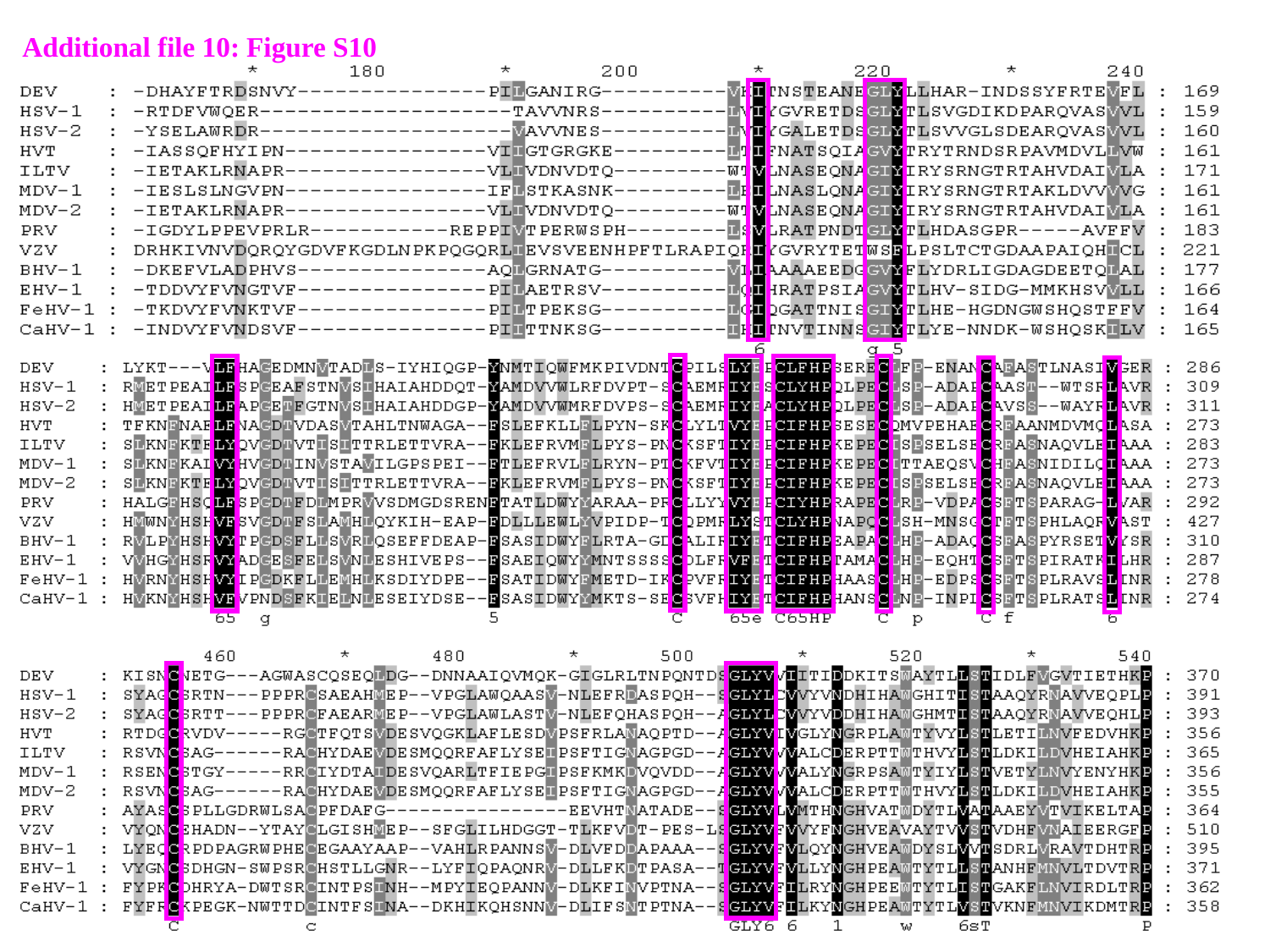

Additional file 10: Figure S10

Supplement: Additional file 10 — Figure S10: Multiple alignments of homologues based on US8 proteins of DEV Clone-03 and other typical strains of the subfamily Alphaherpesvirinae. The conserved domains were indicated by pink boxes. [file 1743-422X-8-200-S10.PPT]
